# Supplementary material for: Factors associated with abnormal left ventricular ejection fraction (decreased or increased) in patients with sepsis in the intensive care unit
Source: PLoS One. 2020 Mar 10;15(3):e0229563. doi: 10.1371/journal.pone.0229563 (PMC7064219; doi:10.1371/journal.pone.0229563)
Supplement: Attachment — Submitted filename: Response to Reviewers.doc [file pone.0229563.s003.doc]

Reviewer(s)' Comments to Author:

**Reviewer #1**

Comments to the Author
Hypothesis- Study was designed to look for risk factors associated with septic cardiomyopathy

In results - Grouping of hyperdynamic and abnormal EF group is not clear, as abnormal EF group includes both hypo and hyperdynamic EF

So interpretation of results is difficult

Need to analyse low EF and hyperdynamic EF separately 🡪 I did it, but I only showed the results of hyperdynamic LV, I added the results of the results of decreased LV EF (table 5). The reason I combined the two groups is because, in each group, mortality tends to increased and similar clinical patterns are seen, but they are not statistically significant individually. Although the results vary individually (esp. in patients with hyperdyabmic LV), patients with abnormal EF have clinical implications because of the increased mortality.

Classification of comorbidities was done based on what? 🡪 In this study, the risk of patients with sepsis was first scored based on SAPS3. Based on previous studies (mainly Sepsis-3, and I added it in the method section and reference 15), patient’s comorbidities (risk factors and major infection focus) were compared using medical records. In addition, patients have recently been residing in nursing homes rather than at home and have come to our home, we further assessed their motor skills and living conditions (bed rest or not). In reviewing echocardiographic results (report and video) and medical records, many patients lived in nursing homes as bed rest state, so I wanted to evaluate whether these factors had adverse effects from venous thrombosis or embolization.

Supplementary material is not in english, so not able to evaluate

Needs plagiarism check 🡪 Although written in Korean, the content is the approval of the local IRB for the restrospective study.

**Reviewer: 2**

Major Comments:

1. Patient selection is unclear in the methods. My read suggests that all patients who were in the ICU with sepsis received an echocardiogram and a cardiology consultation. This seems unusual, as even hospitals with high echocardiography utilization don’t perform echocardiography on 100% of septic patients. I would expect a number of patients admitted with sepsis, and a subset of those patients received a cardiac consultation and echocardiogram. Please clarify. 🡪 I totally agree with you. The original meaning was that it was targeted to “all” patients with sepsis “who performed echocardiography”, not all patients with sepsis. I changed the text to a more clear expression.

2. The authors do not specify how sepsis was determined. Sepsis 3 criteria were disseminated in early 2016. Were these patients categorized according to Sepsis 2, Sepsis 3, a combination? Were they categorized by some screening criteria, or was assignment of sepsis based on a clinical gestalt, medical documentation, or billing? Please clarify. 🡪 The diagnosis and definition of sepsis is so common and mixed, so diagnosis based on exact criteria is important. As you pointed out, it seems that there is a missing reference to the part currently diagnosed accordance with the guidelines. I added it in the method section and reference (15)

1. It is unclear how some of the cardiac patients were excluded. Stress-induced cardiomyopathy can overlap with septic cardiomyopathy. There is disagreement whether septic cardiomyopathy and stress induced cardiomyopathy represent distinct phenotypes. Additionally, the exclusion of antecedent LV dysfunction or restrictive cardiomyopathy likely results in a selection bias, as patients who have had prior echocardiograms are different than those who have not. There is no such thing as ischemic cardiomyopathy. The WHO definition for cardiomyopathy excludes ischemia. The preferred term is ischemic heart disease. Ordinarily, this would just be a pedantic comment, but the inclusion of this term makes me wonder about whether hypertrophic cardiomyopathy is being being conflated with hypertrophic heart—terms clinicians often throw about casually. To be clear, hypertrophic cardiomyopathy is the autosomal dominant disease of excess muscle growth, despite that the term is often used erroneously to describe hypertrophy of hypertension or valvular disease. Regardless, please include a strobe flow diagram that details number of patients included and excluded. 🡪 I added it as a figure 1. (

I already made the flow chart and it didn't seem to make much sense, so I removed it when I submitted this manuscript).

3. The study is retrospective, but the description of the echoes sounds more like a detailed prospectively performed protocol. If the echoes were clinically obtained echoes, please simply state that the echoes were performed clinically in adherence with guidelines by appropriately certified people. Additionally, please clarify that the measurements were clinically obtained by the sonographer or physician, unless the investigators performed additional measurements as part of the research investigation. 🡪 As you pointed out, it may be a “cross-sectional, observational study”, but we retrospectively studied the patients who were hospitalized with sepsis for a period of time. Among them, we included patients who were treated in an intensive care unit and performed echocardiography. The measurements were obtained by sonographer and confirmed by physician (esp. cardiologist) in neart real time. I added it in the method section.

4. As data are non-normally distributed, it is customary to report median and interquartile ranges instead of mean and standard deviation for those data. 🡪 As you mentioned, we reported median (min-max) for non-normally distributed data analyzed by Kruskal-Wallis test. If you want change it as interquartile ranges, please let us know.

5. The authors perform multiple comparisons without an a priori hypothesis. I see scores of comparisons. Please correct for multiple hypothesis testing using Bonferroni or some other similar correction. 🡪 I did it for the significant clinical parameter “female gender”, “Hospital day” and “LAVI”. As a result from Bonferroni correction in female gender, hyperdynamic EF vs. normal EF (p = 0.028), normal vs. reduced EF (p = 0.107), hyperdynamic EF vs. reduced EF (p = 1.000). From the result, there was no statistical significance (All were higher than 0.0167). As a result from Bonferroni correction in hospital days, hyperdynamic EF vs. normal EF (p = 0.005), normal vs. reduced EF (p = 0.615), hyperdynamic EF vs. reduced EF (p = 0.106). As a result from Bonferroni correction in LAVI, hyperdynamic EF vs. normal EF (p = 0.002), normal vs. reduced EF (p = 0.973), hyperdynamic EF vs. reduced EF (p = 0.021). I added it in the statistical analysis section and table, respectively. Strictly speaking, there was no statistical significance in female gender (p>0.0167).

the female gender was found to be a significant factor in the multiple regression analysis, which is clinically meaningful.

6. 48 hours after sepsis is a long time. The authors do not mention accounting for censoring by death within the first 48 hours. Additionally, censoring by death prevents most echocardiographic follow-up. The STROBE diagram might help demonstrate how many patients might have died before having an opportunity to receive echocardiogram. 🡪 Thank you for the good point. Although we also concerned about what you are concerned about, it is difficult to apply in this study. We are continuing our research on patients with sepsis, so we will apply it in the subsequent studies.

7. I would like to see references and comparison to other similar studies in the field. Prior investigations have reported lower incidence of hyperdynamic hypovolemia, and higher incidence of hypocontractility. I suspect this finding is due both to selection criteria, which excluded all patients with antecedent EF<50%, and practice variation where the study population may perhaps receive less volume expansion than those other hospitals. Antecedent fluid receipt may be a more direct predictor of hyperdynamic hypovolemia. Additionally, vasopressors and ventilator receipt could be obvious confounders. 🡪 As you mentioned previously, there are relatively many studies on stress induced CMP, but there is a lack of research on cardiac dysfunction or hyperdynamic LV in patients with sepsis. Therefore, based on the previous studies, this study shows abnormal EF as a new concept, and it definitely showed that abnormal EF is associated with increase in mortality.

We also compared the initial CVP because we thought volume status would affect the EF, but there was no statistical significance. I added some explanation about this in the discussion section with reference.

We also evaluated the use of vasopressors and ventilator receipt, but there was no statistical significance. However, there is a limit to investigate the part (use of vasopressor, what kind of vasopressor, dose, duration, et al. and also ventilator care) more closely, so we omitted that part in the present study.

8. Ventricular function can be affected by so many parameters. Other studies in this field had accounted for vasopressor dose receipt of mechanical ventilation at time of echo, and antecedent fluid receipt. This study should adjust for these confounders.

Minor Comments:

1. Page 8. “...were measured at end-diastole according to the standards established by the American Society of Echocardiography2.” Please omit the 2 🡪 I omitted it. Additionally, the guideline cited by Porter et al. is out of date and has been replaced by a more recent publication. 🡪 I changed it (actually I typed wrong reference).

2. Table 1-3. Group 3 is misspelled. 🡪 I changed it.

Final comments to reviewers:

We have prepared a revision to the best of our ability. First of all, thanks for the valuable comments, my paper is richer and more valuable.
